# Impact of Severe Polypharmacy on Survival in Patients with Heart Failure: A Systematic Review and Meta-Analysis

**DOI:** 10.3390/jcm15218519

**Published:** 2026-11-02

**Authors:** Alejandro Valcuende-Rosique, Clara Bonanad, Sergio Garcia-Blas, Guillermo Barreres, Nora Garcia-Collado, Cristina Monleon-Bonet, Daniela Maidana, Maria Soledad Palacio, Javier Valcuende-Rosique, Francisco Jose Tarazona-Santabalbina

**Affiliations:** 1 Department of Pharmacy, Hospital Padre Jofre, 46017 Valencia, Spain; alexvalcuende@gmail.com; 2 Medical School, Universidad Católica de Valencia San Vicente Mártir, 46001 Valencia, Spain; fjtarazonas@gmail.com; 3 Centro de Investigación Biomédica en Red Fragilidad y Envejecimiento Saludable (CIBERFES), 28029 Madrid, Spain; 4 Department of Medicine, University of Valencia, 46010 Valencia, Spain; 5 Instituto de Investigación Sanitaria INCLIVA, 46010 Valencia, Spain; sergiogarciablas@gmail.com (S.G.-B.); gbarreres@incliva.es (G.B.); nora.garcia.collado@gmail.com (N.G.-C.); cmonleon@incliva.es (C.M.-B.); 6 Centro de Investigación Biomédica en Red de Enfermedades Cardiovasculares (CIBERCV), Instituto de Salud Carlos III, 28029 Madrid, Spain; 7 Department of Cardiology, Hospital Clínico Universitario de Valencia, 46010 Valencia, Spain; 8 Department of Cardiovascular Surgery, Hospital Universitario Virgen Macarena, 41042 Sevilla, Spain; danielamaidana@campus.fmed.uba.ar; 9 Servicio de Cardiología del Centro de Especialidades Médicas de Referencia Nº1 (CEMAR Nº1), Buenos Aires C1427BON, Argentina; solepalacio@hotmail.com; 10 Cardiology Department, Hospital Universitario de Sagunto, Port de Sagunt, 46520 Valencia, Spain; javier.valcuende@gmail.com; 11 Medical Directorate, Hospital La Magdalena, 12004 Castellón de la Plana, Spain

**Keywords:** heart failure, polypharmacy, medication burden, all-cause mortality, meta-analysis

## Abstract

**Background:** Polypharmacy is highly prevalent in patients with heart failure (HF), but the prognostic significance of severe polypharmacy remains uncertain. This systematic review and meta-analysis evaluated its association with all-cause mortality in adults with HF. **Methods:** PubMed, MEDLINE, Embase, Scopus and the Cochrane Library were searched from inception through August 2026. Observational studies assessing severe polypharmacy (the highest medication-burden category in each study; ≥9 to ≥15 medications) and all-cause mortality in adults with HF were eligible. Adjusted hazard ratios (HRs) with 95% confidence intervals (CIs) were pooled using a random-effects model. Leave-one-out sensitivity analyses and post hoc threshold-specific analyses were performed. Risk of bias and certainty of evidence were assessed using the Newcastle–Ottawa Scale (NOS) and GRADE, respectively. The protocol was registered in PROSPERO (CRD420261507517). **Results:** Nine studies included 44,213 patients. Severe polypharmacy was not significantly associated with all-cause mortality (HR 1.04, 95% CI 0.86–1.26; *p* = 0.67; I^2^ = 79%). NOS scores ranged from 6 to 9. Leave-one-out HRs ranged from 0.996 to 1.097, with all 95% CIs including 1. In six studies defining severe polypharmacy as ≥10 medications, the pooled HR was 1.15 (95% CI 1.00–1.32; *p* = 0.054; I^2^ = 37.8%). Certainty of evidence was very low because of serious inconsistency and imprecision. **Conclusions:** No statistically significant association was identified, but very low certainty of evidence precludes firm conclusions, and clinically important associations cannot be excluded. Medication count should be interpreted alongside treatment appropriateness and clinical complexity.

## 1. Introduction

Heart failure (HF) is a major cardiovascular health problem characterized by substantial clinical complexity, frequent hospitalizations and rehospitalizations, and high morbidity and mortality [1,2]. Population aging and the high prevalence of multimorbidity among patients with HF have progressively increased the complexity of HF management. In particular, the coexistence of multiple chronic conditions may complicate HF management and increase the number of prescribed medications, thereby increasing the risk of interactions between diseases and treatments [1,2]. Furthermore, the availability of multiple combinations of therapies that improve HF outcomes has also contributed to an increase in the number of prescribed medications, particularly among older patients and those with a greater comorbidity burden [3,4].

Polypharmacy is therefore particularly common among patients with HF. A systematic review specifically conducted in this population reported a highly variable prevalence of polypharmacy, most commonly defined as the prescription of ≥5 medications, ranging from 17.2% to 99% [5]. However, simply counting the number of medications has important limitations as a measure of therapeutic appropriateness. Numerical definitions of polypharmacy do not necessarily distinguish between appropriate and inappropriate treatments and may not account for patients’ comorbidities and individual therapeutic needs [6]. This issue is particularly relevant in HF because a substantial proportion of the medication burden consists of guideline-directed therapies with demonstrated clinical benefits; thus, a higher number of medications does not necessarily imply greater exposure to potentially harmful treatments [3,7]. Nevertheless, potentially inappropriate prescribing is common in this population. Among older adults hospitalized with HF, potentially inappropriate medications were identified in 61.1% of patients at admission and 64.0% at discharge, with such prescribing patterns being more likely in the presence of polypharmacy [8].

A high medication burden may nevertheless increase treatment complexity and contribute to medication-related problems. In a study of 400 patients hospitalized with HF, potential drug–drug interactions were identified in 68.0% of patients at admission and 88.8% at discharge, while 36.3% had at least one potential interaction classified as major in severity [9]. Greater treatment complexity may also impair medication adherence and persistence. In a cohort of more than 100,000 patients with HF, adherence decreased as regimen complexity increased, with a proportion of days covered of only 42% for triple therapy [10]. These issues may be particularly relevant in older patients with multimorbidity, in whom frailty and polypharmacy frequently coexist [11]. Consequently, severe polypharmacy may simultaneously reflect greater therapeutic complexity and a higher underlying disease burden, making it difficult to determine whether its association with clinical outcomes represents a direct effect of medication exposure or, at least in part, a marker of underlying patient characteristics [2,3].

In recent years, several observational studies have investigated the association between a high medication burden and mortality in patients with HF. Among patients with acute or decompensated HF, severe polypharmacy has been associated with higher mortality, with adjusted hazard ratios (HRs) of 1.48 (95% confidence interval (CI), 1.11–1.99) and 1.16 (95% CI, 1.01–1.33) reported in two cohorts [12,13]. However, other studies have not confirmed this association after adjustment for potential confounders or have reported differences according to HF phenotype, including reduced, mildly reduced, or preserved ejection fraction [14,15]. Available studies have included patients stratified by HF phenotype, as well as populations with acute HF and cohorts of older adults [3,14,15]. Moreover, the criteria used to define severe polypharmacy have been heterogeneous, with thresholds ranging from ≥9 to ≥15 medications [12,13,14,15].

Despite the growing number of observational studies, the association between severe polypharmacy and all-cause mortality in patients with heart failure (HF) remains uncertain. A recent systematic review and meta-analysis investigated the association between polypharmacy and mortality in this population but found no statistically significant association with all-cause mortality [16]. However, broad definitions of polypharmacy encompass patients with substantially different levels of medication burden and may therefore obscure associations specific to those receiving the highest numbers of medications. Whether the absence of a statistically significant association observed for polypharmacy more generally also applies to severe polypharmacy remains unclear. Addressing this question is clinically relevant because patients with the highest medication burden may differ in terms of treatment complexity, comorbidity, and disease severity. Accordingly, a focused synthesis of the available evidence is needed to clarify the association between severe polypharmacy and mortality in patients with HF.

The aim of this meta-analysis was to evaluate the association between severe polypharmacy and all-cause mortality in patients with HF by quantitatively synthesizing the available observational evidence.

## 2. Materials and Methods

### 2.1. The Eligibility Criteria

This systematic review and meta-analysis was prospectively registered in PROSPERO (CRD420261507517) and conducted in accordance with the PRISMA 2020 statement (Supplementary File S1) [17]. The research question was formulated according to the PICOS framework.

Inclusion criteria. Studies were eligible if they met all of the following criteria: (1) observational study design, either prospective or retrospective; (2) inclusion of adults (aged ≥ 18 years) with heart failure (HF), irrespective of HF phenotype or clinical setting, including acute or decompensated HF; (3) assessment of the association between severe polypharmacy and all-cause mortality; (4) definition of severe polypharmacy according to the highest medication-burden category reported in each study, with thresholds of ≥9, ≥10, ≥12, or ≥15 medications; (5) comparison of the severe polypharmacy group with lower medication-burden categories; and (6) availability of effect estimates with corresponding confidence intervals (CIs), or sufficient data to calculate them.

Exclusion criteria. We excluded studies that (1) did not report original data, including systematic reviews and meta-analyses; (2) did not evaluate the association between severe polypharmacy and all-cause mortality; (3) did not report sufficient information on all-cause mortality or provide data to estimate the association of interest; (4) reported populations that overlapped with those of another included study; or (5) were editorials, letters, case reports, case series, protocols, or experimental or animal studies.

No restrictions were imposed on publication year or language.

### 2.2. Information Sources

A systematic literature search was conducted in PubMed, MEDLINE, Embase, Scopus, and the Cochrane Library from database inception through August 2026. To enhance the sensitivity of the search, the reference lists of included studies and relevant publications identified during the review process were manually screened. Additional sources of potentially relevant evidence, including trial registries, grey literature, and reference lists, were also consulted when appropriate.

### 2.3. Search Strategy and Study Identification

The search strategy combined controlled vocabulary and free-text terms related to HF, medication burden, polypharmacy, and mortality. Synonyms and related terms for heart failure, polypharmacy, severe polypharmacy, hyperpolypharmacy, medication burden, number of medications, and mortality were used. Search strategies were adapted to the syntax and controlled vocabulary of each database. The search was conducted without restrictions on publication year or language.

As an example, the PubMed search strategy was:

(“Heart Failure” [Mesh] OR “heart failure” [Title/Abstract] OR “cardiac failure” [Title/Abstract]) AND (“Polypharmacy” [Mesh] OR polypharmacy [Title/Abstract] OR “severe polypharmacy” [Title/Abstract] OR hyperpolypharmacy [Title/Abstract] OR “medication burden” [Title/Abstract] OR “number of medications” [Title/Abstract]) AND (“Mortality” [Mesh] OR mortality [Title/Abstract] OR “all-cause mortality” [Title/Abstract] OR death [Title/Abstract]).

Eligible studies were prospective or retrospective observational studies of adults with HF that evaluated the association between a high medication burden or severe polypharmacy and all-cause mortality. Systematic reviews and meta-analyses, experimental studies, editorials, letters to the editor, protocols, case reports or case series, and publications that did not provide mortality data were excluded.

Two investigators independently screened titles and abstracts to identify potentially eligible studies. Potentially relevant articles then underwent full-text assessment. Disagreements were resolved through discussion and, when necessary, consultation with a third investigator.

### 2.4. Study Selection and Data Extraction

Study selection was conducted in two stages: title and abstract screening followed by full-text assessment, in accordance with the PRISMA 2020 flow diagram. Two investigators independently performed study selection and data extraction using a standardized data collection form.

The following study characteristics were extracted: first author, publication year, country, study design, sample size, population characteristics, HF phenotype, definition and threshold of medication burden, comparator group, follow-up duration, and all-cause mortality. Study characteristics and definitions of medication burden are presented in Table 1.

When multiple adjusted estimates were available, the multivariable model with the greatest degree of adjustment was selected. All included studies reported results as hazard ratios (HRs) with 95% CIs; therefore, HRs were used as the effect measure for the meta-analysis.

### 2.5. Risk of Bias Assessment Methods

Two investigators independently assessed methodological quality using the Newcastle–Ottawa Scale [23], applying review-specific operational criteria. Selection assessment considered cohort representativeness, the origin of comparison groups and medication ascertainment. For these prognostic mortality analyses, the baseline-outcome item was adapted to require participants to be alive when medication exposure was assessed.

One comparability star required adjustment for age and sex; the second required additional adjustment for comorbidities together with at least one measure of heart failure severity, frailty or functional status. Outcome assessment considered mortality ascertainment through clinical or death records, record linkage or adjudication. Follow-up duration and completeness were evaluated separately: the respective stars required a stated mortality horizon or mean/median follow-up of ≥12 months and documented follow-up completeness of ≥90%. Stars were withheld when the available information did not establish that a criterion was met.

Disagreements between investigators were resolved through discussion and, when necessary, consultation with a third investigator.

### 2.6. Statistical Analysis

Quantitative synthesis was performed using Review Manager (RevMan). The effect measure was the HR with its corresponding 95% CI. Effect estimates were harmonized before pooling to ensure a consistent direction of association, such that an HR >1 indicated a higher risk of all-cause mortality associated with severe polypharmacy.

Given the expected clinical and methodological heterogeneity across studies, a random-effects model was used for the primary meta-analysis. Between-study variance (τ^2^) was estimated using restricted maximum likelihood. Results were presented as forest plots showing individual study estimates and the pooled estimate, together with their corresponding 95% CIs.

Statistical heterogeneity was assessed using Cochran’s Q test and quantified using the I^2^ statistic. I^2^ values <25%, 25–50%, and >50% were considered indicative of low, moderate, and substantial heterogeneity, respectively.

### 2.7. Publication Bias Methods

Potential publication bias was assessed by visual inspection of the funnel plot.

Formal statistical tests of funnel plot asymmetry were not performed because of the limited number of included studies, as the reliability of these tests is reduced when relatively few studies are available.

### 2.8. Sensitivity Analysis Methods

Leave-one-out sensitivity analyses were performed by sequentially excluding each study and refitting the random-effects model. Between-study variance was re-estimated using Restricted Maximum Likelihood for each analysis, with Wald 95% confidence intervals. Changes in the pooled HR, τ^2^ and I^2^ were examined. Analyses were implemented in Python using SciPy 1.17.0.

A post hoc exploratory analysis stratified studies by the medication-count threshold defining severe polypharmacy (≥9, ≥10, ≥12 or ≥15 medications). The six studies using the ≥10 threshold were pooled using a random-effects model with restricted maximum likelihood estimation and Wald 95% confidence intervals. Strata containing one study were presented individually, without formal between-subgroup comparisons.

### 2.9. Certainty of the Evidence

The certainty of the evidence for the outcome of all-cause mortality was assessed using the Grading of Recommendations Assessment, Development and Evaluation (GRADE) approach [24].

The assessment considered the five GRADE domains: risk of bias, inconsistency, indirectness, imprecision, and publication bias. Overall certainty of the evidence was classified as high, moderate, low, or very low according to the GRADE framework.

## 3. Results

### 3.1. Study Selection

**Figure 1:**
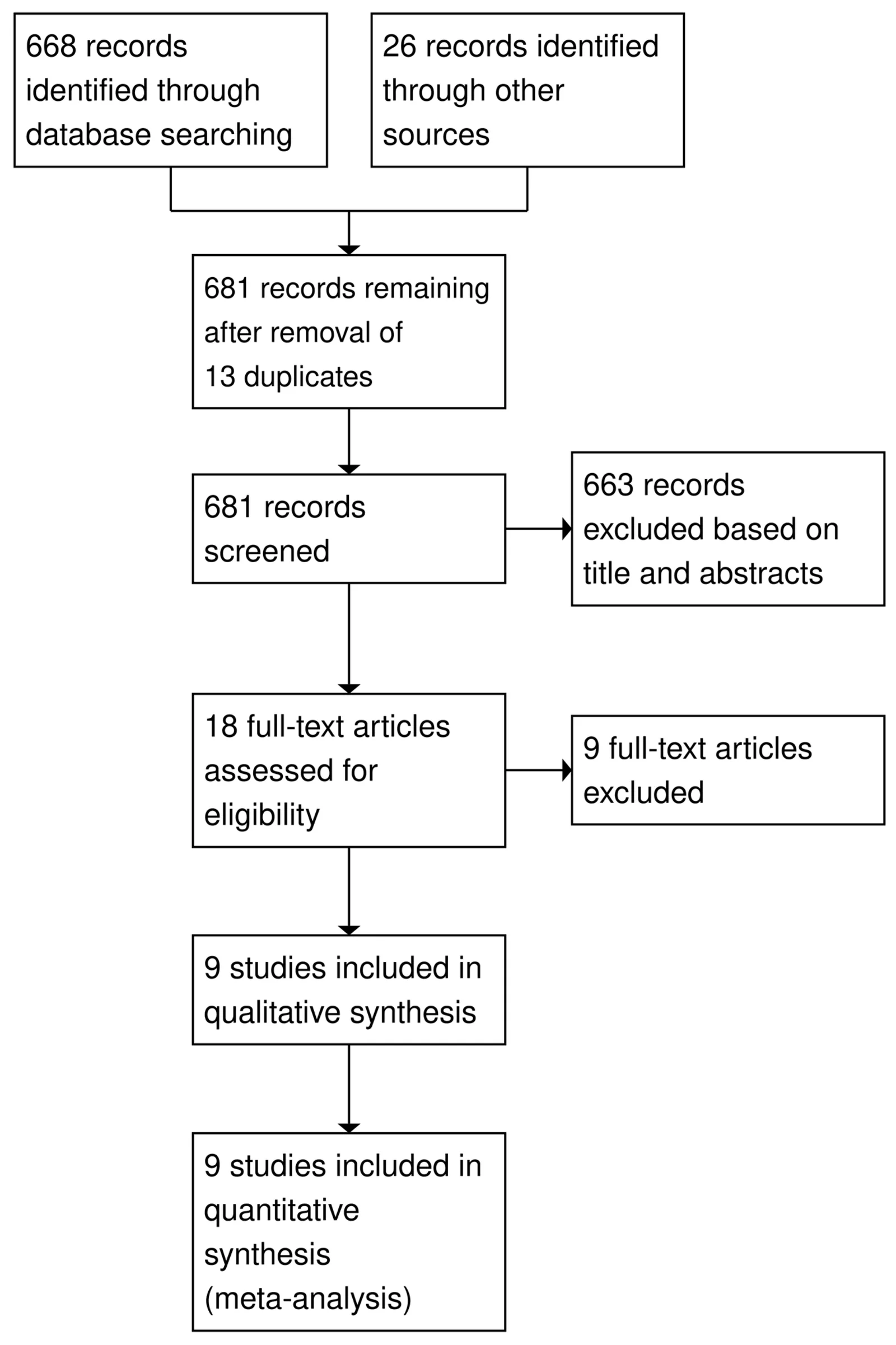
Flow diagram depicting the study selection process.

The study selection process is shown in Figure 1. The literature search and additional searches of trial registries, grey literature databases, and reference lists identified 681 records. After removal of duplicates and screening of titles and abstracts, 18 articles underwent full-text assessment for eligibility. Of these, 9 were excluded because they did not meet the prespecified inclusion criteria. Ultimately, 9 studies were included in the systematic review and meta-analysis.

### 3.2. Study Characteristics

The main characteristics of the included studies are summarized in Table 1. Overall, 9 observational studies comprising 44,213 patients with HF were included [12,13,14,15,18,19,20,21,22]. Studies were conducted in Asia, Europe, the Americas, and international populations and included different HF phenotypes, including reduced, mildly reduced, or preserved ejection fraction, as well as patients with acute or decompensated HF. Sample sizes ranged from 660 to 18,030 patients. The definition of severe polypharmacy varied across studies, with thresholds for the highest medication-burden category ranging from ≥9 to ≥15 medications. All studies evaluated all-cause mortality.

### 3.3. Risk of Bias Assessment

Methodological quality was assessed using the Newcastle–Ottawa Scale with review-specific operational criteria. Scores ranged from 6 to 9, with eight studies classified as high quality and one as moderate quality (Table 2).

### 3.4. Meta-Analysis

**Figure 2:**
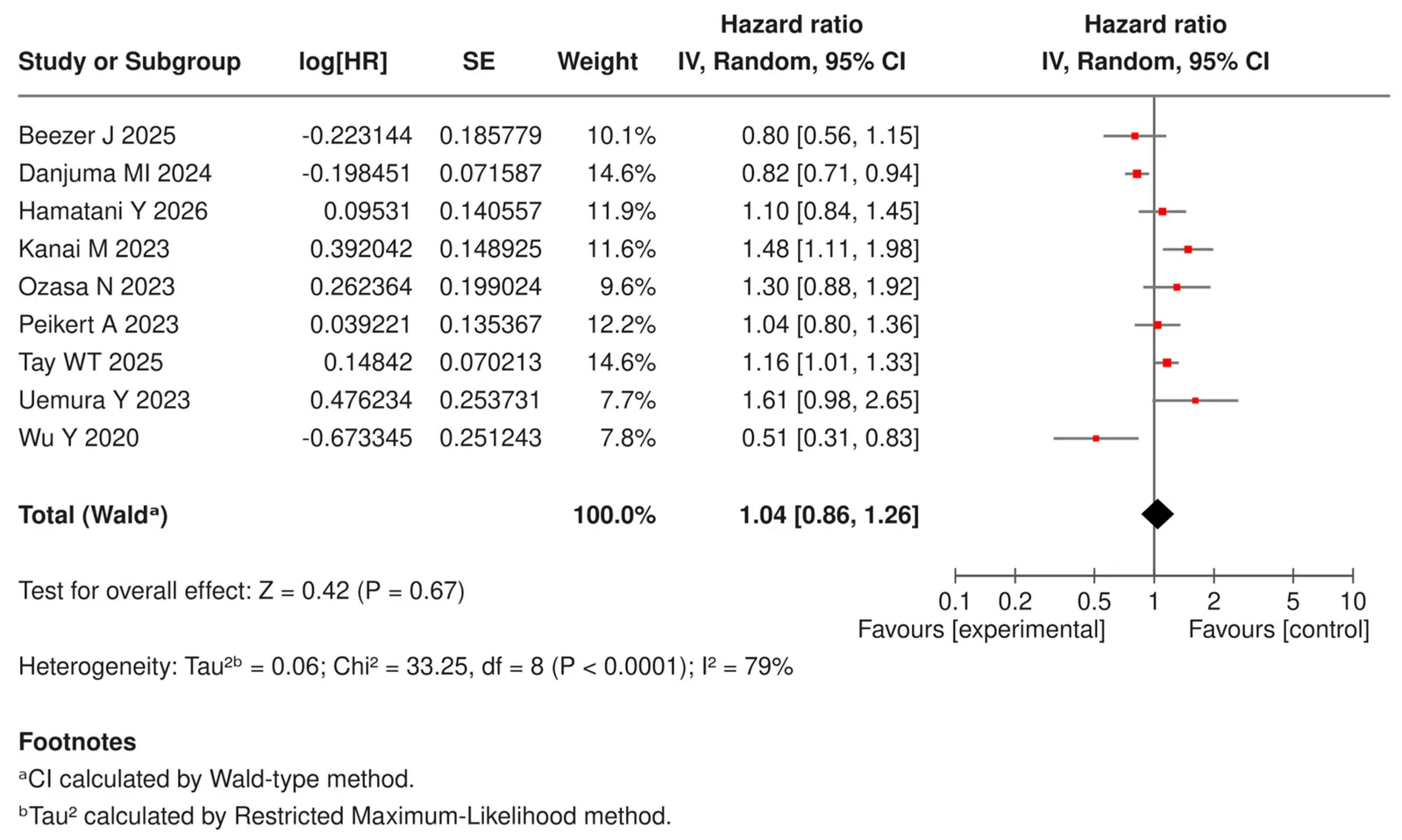
Forest plot of the association between severe polypharmacy and all-cause mortality in patients with heart failure. Red squares represent individual study estimates, with their size proportional to study weight; horizontal lines indicate 95% confidence intervals. The black diamond represents the pooled estimate and its 95% confidence interval, highlighted in bold in the corresponding row. CI: confidence interval. HR: hazard ratio [12,13,14,15,20,21,22,23,24].

The meta-analysis evaluated the association between severe polypharmacy and all-cause mortality in patients with HF. The results are shown in Figure 2.

### 3.5. Severe Polypharmacy and All-Cause Mortality

All 9 studies contributed to the primary meta-analysis, comprising a total of 44,213 patients [18,19,20,21,22,25,26,27,28]. Severe polypharmacy was not significantly associated with a higher risk of all-cause mortality compared with the lower medication-burden categories (HR, 1.04; 95% CI, 0.86–1.26; *p* = 0.67). Substantial heterogeneity was observed across studies (I^2^ = 79%).

### 3.6. Sensitivity Analysis

Across the nine leave-one-out analyses, pooled HRs ranged from 0.996 to 1.097, and all 95% confidence intervals included 1. Heterogeneity remained substantial (I^2^ = 70.5–83.0%). Excluding Wu et al. [22] yielded the highest pooled estimate (HR 1.097; 95% CI 0.931–1.294) and the lowest heterogeneity (I^2^ = 70.5%). Thus, no individual exclusion established a statistically significant association, although substantial between-study variability persisted (Supplementary File S2).

Among the six studies using a threshold of ≥10 medications, the pooled HR was 1.149 (95% CI 0.998–1.322; *p* = 0.054), with I^2^ = 37.8%. The ≥9, ≥12 and ≥15 thresholds were each represented by a single study and are displayed individually in the supplementary forest plot (Supplementary File S3).

### 3.7. Publication Bias

**Figure 3:**
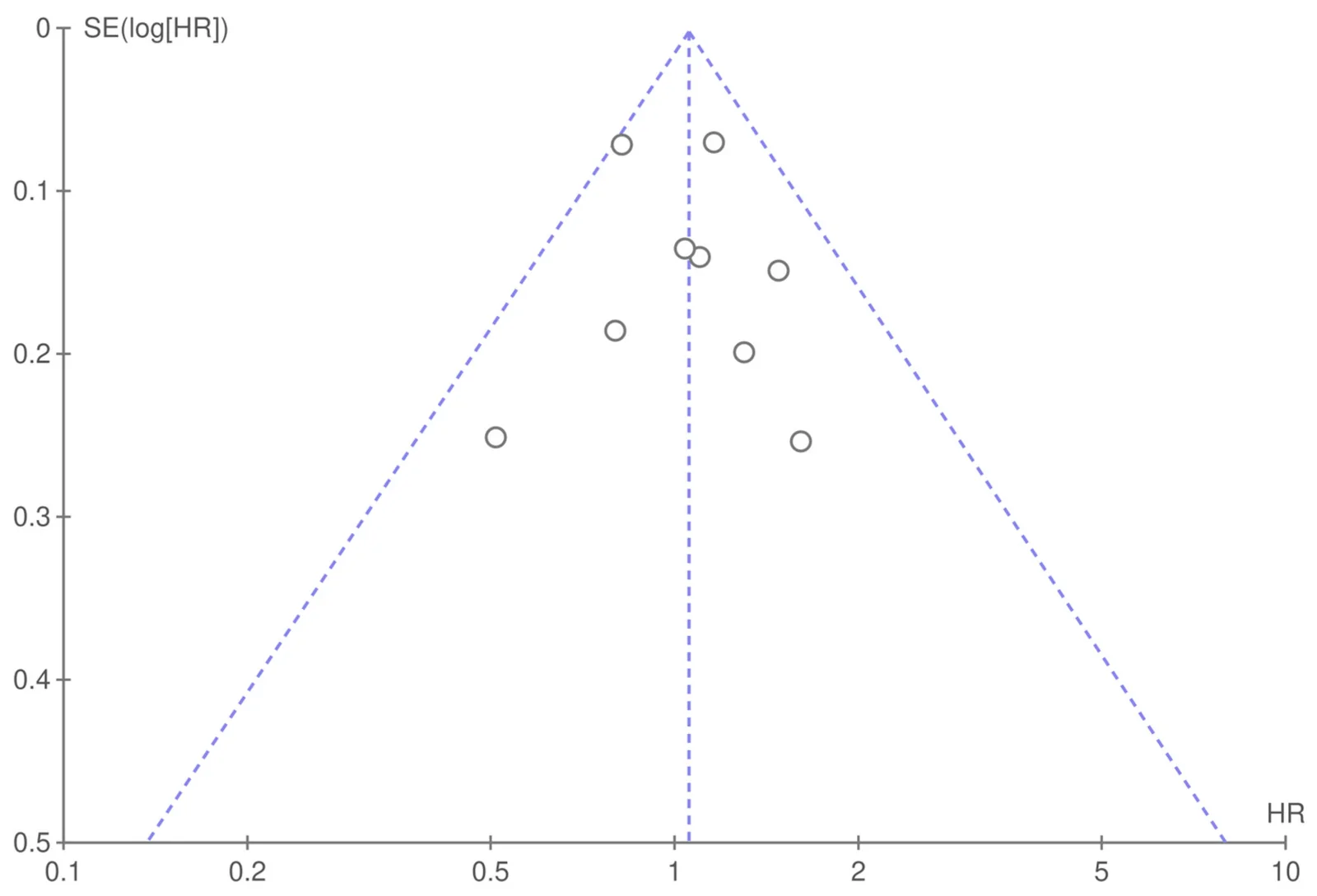
Funnel plot for the assessment of publication bias in the meta-analysis of the association between severe polypharmacy and all-cause mortality. Circles represent individual studies, the vertical dotted line indicates the pooled effect estimate, and the diagonal dotted lines represent the pseudo-95% confidence limits. HR: hazard ratio.

Visual inspection of the funnel plot showed an overall symmetric distribution of studies around the pooled estimate, with no substantial evidence of asymmetry (Figure 3). All studies were located within the expected limits of the funnel, with no relevant visual indication of publication bias. However, the funnel plot should be interpreted cautiously given the limited number of included studies.

### 3.8. GRADE Assessment

According to the GRADE framework, the certainty of evidence for the association between severe polypharmacy and all-cause mortality was very low owing to serious inconsistency and imprecision. Substantial unexplained heterogeneity was observed (I^2^ = 79%). Imprecision was considered serious because the pooled estimate (HR 1.04; 95% CI 0.86–1.26) was compatible with a lower mortality hazard, no association and a potentially clinically important increase in mortality hazard, limiting confidence in the magnitude and direction of the association (Table 3).

## 4. Discussion

This systematic review and meta-analysis evaluated the association between severe polypharmacy and all-cause mortality in patients with heart failure. Across the nine observational studies included [12,13,14,15,18,19,20,21,22], severe polypharmacy was not significantly associated with all-cause mortality, with a pooled HR of 1.04 (95% CI, 0.86–1.26). Leave-one-out sensitivity analyses yielded similar pooled estimates (HR range, 0.996–1.097), with all 95% confidence intervals including 1, supporting the stability of the pooled estimate across individual study exclusions.

These results suggest that medication count alone may be insufficient to characterize mortality risk in patients with heart failure. Although some individual studies have reported an association between a greater medication burden and mortality, these associations may be attenuated after accounting for patients’ clinical characteristics, such as comorbidity burden or disease severity. Multimorbidity is closely associated with prognosis in heart failure and may simultaneously contribute to a greater medication burden [1,2]. Moreover, the use of guideline-directed medical therapy means that a higher medication count may, at least in part, reflect therapeutic optimization rather than greater exposure to potentially harmful treatments [3,4,25].

Substantial heterogeneity was observed across studies (I^2^ = 79%). Differences in heart failure phenotype, clinical setting, follow-up duration, patient characteristics, and the thresholds used to define severe polypharmacy may have contributed to the variability in effect estimates. Heterogeneity was lower among the six studies using the ≥10-medication threshold (I^2^ = 37.8%); however, this exploratory finding does not establish that threshold differences explain the overall heterogeneity. Furthermore, medication count does not fully capture regimen complexity or overall treatment burden. Highly complex medication regimens have been described in older patients with heart failure and may impair adherence and increase the risk of adverse events [26].

The absence of a significant association in the pooled analysis does not imply that a high medication burden is clinically irrelevant. Medication counts alone cannot distinguish between appropriate treatments, including guideline-directed therapies, and potentially inappropriate or unnecessary medications. Potentially inappropriate medications are common among older adults hospitalized with heart failure [8,27]. Similarly, a high prevalence of polypharmacy, potentially inappropriate medications, and therapeutic competition has been reported in patients with heart failure with preserved ejection fraction, highlighting the complexity that may remain concealed when medication exposure is reduced solely to the number of drugs [28].

Conversely, a greater medication burden may affect treatment adherence and persistence. Treatment regimen complexity represents a potential barrier to adherence to recommended therapies, particularly among older patients [9,26]. Thus, the clinical effect of polypharmacy may depend on the balance between the benefits of indicated treatments and the problems associated with treatment regimen complexity. This concept is consistent with recent studies showing that total medication count may simultaneously encompass guideline-directed therapies associated with improved outcomes and potentially inappropriate medications associated with adverse outcomes in certain patients [4,29].

The relationship between polypharmacy, frailty, and prognosis also warrants consideration. Frailty is common among patients with heart failure and is associated with a greater likelihood of polypharmacy [10,30]. In this context, polypharmacy may partly reflect greater clinical vulnerability and disease burden, complicating the interpretation of its independent association with mortality. This potential overlap among medication burden, multimorbidity, and frailty may have contributed, at least in part, to the heterogeneity observed across studies.

These findings have implications for the interpretation of polypharmacy as a prognostic marker. Rather than considering medication count alone, assessment of medication burden should incorporate treatment indication, appropriateness, dose, duration, potential drug–drug interactions, comorbidities, and the patient’s functional status. Likewise, factors such as orthostatic hypotension, treatment-related fall risk, and anticholinergic burden may provide additional information for characterizing medication burden, although these factors have received limited attention in studies of patients with heart failure [31,32]. In this regard, medication review and optimization strategies, including deprescribing when clinically indicated, should balance treatment burden and tolerability with the preservation of therapies that provide prognostic benefit [4,33,34,35]. Recent studies further highlight the importance of assessing medication appropriateness and adherence together [36], with polypharmacy associated with non-adherence in ambulatory patients with heart failure [37].

Finally, these findings should be interpreted in the context of the available evidence on polypharmacy in heart failure. A recent meta-analysis of polypharmacy also found no significant association with all-cause mortality (HR, 1.05; 95% CI, 0.88–1.25) [16]. Our study builds on this evidence by specifically examining whether this lack of a statistically significant association also applies to the highest categories of medication burden, defined by thresholds ranging from ≥9 to ≥15 medications. The pooled estimate was close to the null (HR, 1.04; 95% CI, 0.86–1.26), suggesting that severe polypharmacy, as defined by medication count alone, was not significantly associated with all-cause mortality in the pooled analysis. However, substantial between-study heterogeneity and the very low certainty of the evidence preclude concluding that severe polypharmacy has no prognostic effect. These findings highlight the need to distinguish medication quantity from treatment appropriateness, regimen complexity, and underlying clinical severity when evaluating the prognostic significance of medication burden in patients with heart failure.

This meta-analysis has several limitations that should be considered when interpreting the findings. First, all analyses of medication burden were observational, limiting causal inference. Although most studies had high Newcastle–Ottawa Scale (NOS) scores, these do not guarantee adequate control of confounding. Differences in covariate adjustment and residual confounding by multimorbidity, heart failure severity, frailty, functional status and guideline-directed medical therapy may have attenuated or exaggerated the observed associations. Therefore, the pooled estimate should not be interpreted as evidence that medication burden has no effect on mortality. Restrictive eligibility criteria in some cohorts may also limit generalisability, while incomplete reporting of follow-up limits the assessment of potential attrition bias. Second, substantial clinical and methodological heterogeneity existed across studies, including differences in heart failure phenotypes, clinical settings, follow-up duration, thresholds used to define severe polypharmacy (≥9 to ≥15 medications), and comparator groups. These differences may limit the comparability of individual effect estimates and complicate the interpretation of pooled results. Third, the limited number of studies restricted our ability to explore sources of heterogeneity reliably. Threshold-specific analyses were exploratory, with only one study available for each threshold other than ≥10 medications. Furthermore, medication counts do not capture potentially relevant aspects of pharmacotherapy, such as treatment appropriateness, dose, duration, indication or drug–drug interactions. Finally, although visual inspection of the funnel plot did not reveal evident asymmetry, the small number of studies limits the ability to reliably detect potential publication bias.

Future research should prioritize large, prospective multicenter studies using standardized definitions of severe polypharmacy and consistent adjustment for relevant clinical confounders. Assessing medication appropriateness, treatment complexity, and adherence alongside medication count may help clarify the independent prognostic significance of severe polypharmacy. Individual participant data meta-analyses could further harmonize exposure definitions and investigate sources of heterogeneity.

## 5. Conclusions

In this meta-analysis, severe polypharmacy was not significantly associated with all-cause mortality in patients with heart failure. However, substantial heterogeneity and very low certainty of evidence preclude firm conclusions, and a clinically important association cannot be excluded. Medication count should be interpreted alongside treatment appropriateness, regimen complexity, comorbidities and heart failure severity. Further prospective studies with consistent exposure definitions, comparable reference groups and adequate control of confounding are needed to clarify the prognostic significance of severe polypharmacy in patients with heart failure.

## Figures and Tables

**Table 1:** Characteristics of studies included in the meta-analysis.

Study (Year)	Country/ Region	Design/ Data Source	N	HF Population/Phenotype	Age	Medication Burden Definition	Comparison	Follow-Up	Outcome
Beezer J et al. (2025) [14]	United Kingdom	Observational study	660	HF; HFrEF and HFpEF subgroups	Mean 76.1 years	1–4; 5–9; ≥10 medications	≥10 vs. 5–9	Median 2.9 years	All-cause mortality
Danjuma MI et al. (2024) [18]	Qatar	Prospective observational study	7354	HF; phenotype not differentiated	Median 59 years (IQR 48–71)	≤4; 5–8; ≥9 medications	≥9 vs. ≤4	Up to 1.6 years	All-cause mortality
Hamatani Y et al. (2026) [15]	International	Prespecified analysis	6001	HFmrEF/HFpEF	72 ± 10 years	≤4; 5–9; ≥10 medications	≥10 vs. ≤4	2.7 years	All-cause mortality
Kanai M et al. (2024) [12]	Japan	Prospective observational study	884	Acute decompensated HF	Median 81 years	<5, 5–9, ≥10 medications	≥10 vs. <5	2 years	All-cause mortality
Ozasa N et al. (2023) [13]	Japan	Observational study	2578	Acute decompensated HF	Median 77 years (IQR 69–84)	Q1 ≤5; Q2 6–8; Q3 9–11; Q4 ≥12 medications	≥12 vs. ≤5	1 year	All-cause mortality
Peikert A et al. (2023) [19]	International	Post hoc analysis	6263	HFmrEF/HFpEF; LVEF > 40%	>40 years	<5; 5–9; ≥10 medications	≥10 vs. <5	NR	All-cause mortality
Tay WT et al. (2025) [20]	International	Prospective observational study	18,030	Acute HF; HFrEF/HFmrEF/HFpEF	66 ± 14 years	<5, 5–9, ≥10 non-HF medications	≥10 vs. <5	1 year	All-cause mortality
Uemura Y et al. (2024) [21]	Japan	Observational study	682	Acute HF in octogenarians/nonagenarians	≥80 years	<10 vs. ≥10 medications at discharge	≥10 vs. <10	NR	All-cause mortality
Wu Y et al. (2021) [22]	Americas	Retrospective observational study	1761	HFpEF; LVEF ≥ 45%	Median 72 years (IQR 64–79)	<5; 5–9; 10–14; ≥15 medications	≥15 vs. <5	NR	All-cause mortality

HF: heart failure; HFpEF: heart failure with preserved ejection fraction; HFmrEF: heart failure with mildly reduced ejection fraction; HFrEF: heart failure with reduced ejection fraction; LVEF: left ventricular ejection fraction; IQR: interquartile range; NR: not reported.

**Table 2:** Methodological quality of the included studies according to the Newcastle–Ottawa Scale.

Study	Selection	Comparability	Outcome	Interpretation
Beezer J et al. (2025) [14]	✭ ✭ ✭ ✭	✭ ✭	✭ ✭ ✭	High quality
Danjuma MI et al. (2024) [18]	✭ ✭ ✭ ✭	✭ ✭	✭ ✭ ✭	Moderate quality
Hamatani Y et al. (2026) [15]	✭ ✭ ✭ ✭	✭ ✭	✭ ✭ ✭	High quality
Kanai M et al. (2024) [12]	✭ ✭ ✭ ✭	✭ ✭	✭ ✭ ✭	High quality
Ozasa N et al. (2023) [13]	✭ ✭ ✭ ✭	✭ ✭	✭ ✭ ✭	High quality
Peikert A et al. (2023) [19]	✭ ✭ ✭ ✭	✭ ✭	✭ ✭ ✭	High quality
Tay WT et al. (2025) [20]	✭ ✭ ✭ ✭	✭ ✭	✭ ✭ ✭	High quality
Uemura Y et al. (2024) [21]	✭ ✭ ✭ ✭	✭ ✭	✭ ✭ ✭	High quality
Wu Y et al. (2021) [22]	✭ ✭ ✭ ✭	✭ ✭	✭ ✭ ✭	High quality

Stars represent the assessment criteria of the Newcastle–Ottawa Scale: selection (maximum 4 points), comparability (maximum 2 points), and outcome (maximum 3 points). Green stars indicate fulfilled criteria (1 point), whereas red stars indicate unfulfilled criteria (0 points). Higher scores indicate better methodological quality.

**Table 3:** GRADE assessment of the certainty of evidence for all-cause mortality.

Certainty Assessment							№ of Patients	Effect	Certainty	Importance
№ of Studies	Study Design	Risk of Bias	Inconsistency	Indirectness	Imprecision	Other Considerations	Total Patients	Effect Estimate (95% CI)		
All-cause mortality according to polypharmacy status										
9	non-randomised studies	not serious	serious ^a^	not serious	serious ^b^	none	44.213	1.04 [0.86–1.26]	⊕◯◯◯ Very low ^a^	CRITICAL

^a^: Possible heterogeneity, ^b^: 95% CI included no association and a potentially important increase in mortality Hazard. The symbols represent the certainty of evidence according to the GRADE framework. One filled circle and three empty circles indicate very low certainty of evidence. CI: confidence interval.

## Data Availability

The original contributions presented in this study are included in the article. Further inquiries can be directed to the corresponding authors.

## Supplementary Materials

The following supporting information can be downloaded at: https://www.mdpi.com/article/10.3390/jcm15218519/s1, Supplementary File S1: PRISMA 2020 checklist, indicating the location of each reporting item within the manuscript. Supplementary File S2: Leave-one-out sensitivity analysis of all-cause mortality, reporting pooled hazard ratios (HRs), 95% confidence intervals (CIs), *p*-values, between-study variance (τ^2^), and heterogeneity (I^2^) after sequential exclusion of each study. Supplementary File S3: Forest plot of the association between severe polypharmacy and all-cause mortality in patients with heart failure, stratified by medication-count threshold (≥9, ≥10, ≥12, and ≥15 medications). Squares represent individual study estimates, horizontal lines indicate 95% CIs, and the diamond represents the pooled estimate for the ≥10-medication subgroup. Strata containing one study are presented individually.

## Abbreviations

The following abbreviations are used in this manuscript:

CI	Confidence interval
HF	Heart failure
HR	Hazard ratio
