# Supplementary material for: Impact of Severe Polypharmacy on Survival in Patients with Heart Failure: A Systematic Review and Meta-Analysis
Source: J Clin Med. 2026 Nov 2;15(21):8519. doi: 10.3390/jcm15218519 (PMC13764361; doi:10.3390/jcm15218519)
Supplement: Supplementary file 1 [file jcm-15-08519-s001.zip › Supplementary File S2.pdf]

Supplementary File S2. Leave-one-out sensitivity analysis of all-cause mortality.

Study omitted	N	HR (95% CI)	P value	τ^2	I^2 (%)
**None (all nine studies)**	**9**	**1.042 (0.860–1.263)**	**0.674**	**0.061**	**79.0**
Beezer 2025	8	1.074 (0.873–1.320)	0.501	0.064	80.8
Danjuma 2024	8	1.086 (0.885–1.333)	0.429	0.058	72.9
Hamatani 2026	8	1.034 (0.825–1.295)	0.771	0.078	83.0
Kanai 2024	8	0.996 (0.824–1.203)	0.964	0.049	75.5
Ozasa 2023	8	1.017 (0.824–1.257)	0.873	0.068	81.9
Peikert 2023	8	1.042 (0.831–1.307)	0.723	0.079	83.0
Tay 2025	8	1.024 (0.815–1.286)	0.841	0.078	78.2
Uemura 2024	8	1.006 (0.829–1.221)	0.951	0.056	79.1
Wu 2021	8	1.097 (0.931–1.294)	0.269	0.035	70.5

N, number of studies; HR, hazard ratio; CI, confidence interval; τ^2, between-study variance. Each exclusion retains eight studies. Models used inverse-variance random effects with Restricted Maximum Likelihood re-estimation of τ^2 and normal/Wald confidence intervals. I^2 was calculated from τ^2 and the typical within-study variance, consistently with the primary analysis.
