# Supplementary material for: Impact of Severe Polypharmacy on Survival in Patients with Heart Failure: A Systematic Review and Meta-Analysis
Source: J Clin Med. 2026 Nov 2;15(21):8519. doi: 10.3390/jcm15218519 (PMC13764361; doi:10.3390/jcm15218519)
Supplement: Supplementary file 1 [file jcm-15-08519-s001.zip › Supplementary File S3.pdf]

Supplementary File S3. Association between severe polypharmacy and all-cause mortality in heart failure stratified by medication threshold

Study or Subgroup	log[HR]	SE	Weight	Hazard ratio IV, Random, 95% CI
**1.1 ≥9 medications**				
Danjuma MI 2024	-0.198451	0.071587	-	0.82 [0.71, 0.94]
**1.2 ≥10 medications**				
Beezer J 2025	-0.223144	0.185779	11.3%	0.80 [0.56, 1.15]
Hamatani Y 2026	0.095310	0.140557	16.7%	1.10 [0.84, 1.45]
Kanai M 2024	0.392042	0.148925	15.5%	1.48 [1.11, 1.98]
Peikert A 2023	0.039221	0.135367	17.5%	1.04 [0.80, 1.36]
Tay WT 2025	0.148420	0.070213	32.1%	1.16 [1.01, 1.33]
Uemura Y 2024	0.476234	0.253731	6.8%	1.61 [0.98, 2.65]
**Subtotal (Wald)**			**100.0%**	**1.149 [0.998, 1.322]**
Test for overall effect: Z = 1.93 (P = 0.054)				
Heterogeneity: Tau² = 0.011; Chi² = 9.11, df = 5 (P = 0.105); I² = 37.8%				
**1.3 ≥12 medications**				
Ozasa N 2023	0.262364	0.199024	-	1.30 [0.88, 1.92]
**1.4 ≥15 medications**				
Wu Y 2021	-0.673345	0.251243	-	0.51 [0.31, 0.83]

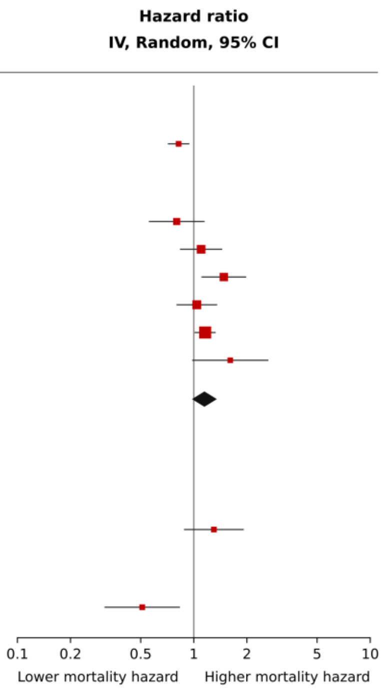

Footnotes

CI calculated by the Wald-type method. Tau² calculated by the restricted maximum likelihood method.
Weights refer to the ≥10-medication stratum. Single-study strata are displayed without pooling.
The pooled HR and CI are shown to three decimal places. Comparator groups differ across studies.
Exploratory stratification by the threshold defining severe polypharmacy; no formal between-subgroup test.

Squares represent study-specific adjusted hazard ratios, with horizontal lines indicating 95% confidence intervals. The diamond represents the pooled estimate for the six studies using the ≥10-medication threshold. Strata containing one study are presented individually.
